# Circ_16601 facilitates Hippo pathway signaling via the miR-5580-5p/FGB axis to promote my-CAF recruitment in the TME and LUAD progression

**DOI:** 10.1186/s12931-023-02566-4

**Published:** 2023-11-12

**Authors:** Jie Zhou, Peiwei Li, Xiaogang Zhao, Yuanhao Zhao, Junwen Luo, Yupeng Deng, Ning Jiang, Zhaohua Xiao, Wenhao Zhang, Yongjia Zhou, Jiangfeng Zhao, Peichao Li, Yuliang Li, Zhongxian Tian

**Affiliations:** 1 Department of Thoracic Surgery, The Second Hospital, Cheeloo College of Medicine, Shandong University, Jinan, Shandong China; 2 Institute of Medical Sciences, The Second Hospital, Cheeloo College of Medicine, Shandong University, Jinan, Shandong China; 3 Shandong Province Key Laboratory of Fundamental Research and Clinical Translation in Thoracic Cancer, Jinan, Shandong China; 4 Department of Pathology, The Second Hospital, Cheeloo College of Medicine, Shandong University, Jinan, Shandong China; 5 Department of Interventional Medicine, The Second Hospital, Cheello College of Medicine, Shandong University, Jinan, Shandong China; 6 Institute of Interventional Oncology, Shandong University, Jinan, Shandong China

**Keywords:** Circ_16601, miR-5580-5p, LUAD, FGB, HIPPO pathway, My-CAF

## Abstract

**Background:**

Lung cancer represents a significant public health issue in China, given its high incidence and mortality rates. Circular RNAs (circRNAs) have been recently proposed to participate in the development and progression of tumors. Nevertheless, their particular roles in the pathogenesis of lung adenocarcinoma (LUAD), the tumor microenvironment (TME), and the underlying molecular mechanisms are still not well understood.

**Methods:**

High-throughput sequencing was used to analyze the circRNAs expression profiles in 7 pairs of human LUAD tissues. shRNA was used to knockdown the YAP1 and FGB genes. RNA sequencing and RT-qPCR were performed to classify the regulatory effects of circ_16601 in LUAD cells. The progression effect of circ_16601 on lung cancer was investigated in vitro and in vivo.

**Results:**

The circ_16601 is significantly elevated in LUAD tissues compared to adjacent normal lung tissues, and its high expression is positively associated with poor prognosis in LUAD patients. Additionally, circ_16601 overexpression promotes LUAD cell proliferation in vitro and increases xenograft tissue growth in mice in vivo; circ_16601 also could recruit fibroblasts to cancer associate fibroblasts. Mechanistically, circ_16601 can directly bind to miR-5580-5p, preventing its ability to degrade FGB mRNA and enhancing its stability. Subsequently, circ_16601 promotes the activation of the Hippo pathway in a YAP1-dependent manner, leading to LUAD progression.

**Conclusions:**

Our findings shed valuable insights into the regulatory role of circ_16601 in LUAD progression and highlight its potential as a diagnostic and therapeutic target in LUAD. Overall, this study provides theoretical support to improve the prognosis and quality of life of patients suffering from this devastating disease.

**Supplementary Information:**

The online version contains supplementary material available at 10.1186/s12931-023-02566-4.

## Background

Lung cancer is a pressing public health concern in China, characterized by increasing incidence and high mortality rates [1, 2]. Among lung cancer cases, lung adenocarcinoma (LUAD) represents the predominant type, accounting for approximately 85% of cases in China [3]. Although the implementation of lung cancer screening has reduced the number of patients with advanced tumors [4]. However, prolonging the 5-year survival rate and disclosing the molecular mechanisms underlying the development and progression of LUAD remain significant challenges. Therefore, it is crucial to identify new potential therapeutic targets and to reveal the signaling pathways that regulate LUAD development. Targeted therapy has emerged as a promising approach for solid tumors and has shown to improve the prognosis of LUAD patients [5–7]. However, selecting the appropriate target for individual patients is challenging, and the development of drug resistance remains a major obstacle leading to poor prognosis [8, 9]. Studies have linked the development of resistance to targeted therapy or monotherapy with cancer-associated fibroblasts (CAF) [10–12]. Despite ongoing efforts to develop drugs targeting tumor-associated fibroblasts, progress has been slow [13, 14]. Hence, understanding the relationship between tumor cells and CAF within the context of tumor tissue heterogeneity could contribute to the development of comprehensive therapies for lung cancer.

Recent studies have explored the regulatory role of circular circRNAs in cancer [15]. CircRNAs, a class of noncoding RNAs with closed-loop structures, have been implicated in tumorigenesis and progression in LUAD [16, 17]. Several studies have highlighted the functional roles of circRNAs, such as hsa_circ_0017109 and circRNA-002178 in LUAD. These circRNAs function by acting as miRNA sponges, modulating gene expression at the translation effective or RNA splicing mediated RNA stability, and serving as protein scaffolds [16, 18]. Furthermore, circRNAs can be packaged in exosomes to regulate myofibroblast-like cancer-associated fibroblast (my-CAF) formation in the tumor microenvironment and cancer progression [19, 20]. However, the specific functional implications of circ_16601 (circ_ID: hsa_circ_0016601), which is generated from back-splicing the exons 1–10 of DNAH14 gene with a length of 1140 nt, in the LUAD tumor microenvironment and LUAD progression remains unclear. Therefore, elucidating the functions of circ_16601 in regulating crucial signaling pathways and my-CAF-associated cancer progression is essential to comprehend the circ_16601-mediated oncogenic progression of LUAD.

The *FGB* gene, encoding the fibrinogen beta chain, has been associated with LUAD [21]. Elevated *FGB* gene expression and fibrinogen levels have been linked to an increased risk of developing lung cancer and poorer prognosis in lung cancer patients [22]. Several studies have explored the role of circRNAs in regulating FGB expression. One study found that a circRNA named hsa_circ_0012138 was significantly upregulated in renal fibrosis and that it serves as a miR-7682-3p sponge to upregulate *FGB* expression (23). Herein, we demonstrate that circ_16601 promotes LUAD progression and my-CAF recruitment in the tumor microenvironment by upregulating *FGB* expression. These findings suggest that circRNAs may regulate *FGB* expression and contribute to the development and progression of LUAD. Therefore, targeting circRNAs that regulate *FGB* expression may represent a novel therapeutic approach for the treatment of LUAD.

The Hippo signaling pathway is an evolutionarily conserved pathway governing cell proliferation, apoptosis, and differentiation [24]. Hippo pathway dysregulation has been observed in various human cancers, including LUAD, and plays a critical role in tumor initiation, progression, and metastasis [25–27]. Recent studies have highlighted the involvement of circRNAs in the regulation of FGB and the Hippo pathway in LUAD [18, 28, 29]. For example, circ_0072083 has been shown to promote LUAD cell proliferation, migration, and invasion by modulating *FGB* expression. Similarly, circ_0067741 has been shown to inhibit LUAD cell proliferation and invasion by ordering the Hippo signaling pathway [30]. In this project, we revealed that circ_16601 activated the Hippo pathway by increasing *FGB* expression and ultimately contributed to LUAD progression.

In this study, we demonstrated that a significant upregulation of circ_16601 in LUAD tissues and cell lines. Functional analysis showed that abnormally high levels of circ_16601 promote LUAD progression in vitro and in vivo. Furthermore, elevated expression of circ_16601 was positively associated with a poor prognosis in LUAD patients and facilitated the recruitment of fibroblasts for induction into my-CAFs. Mechanistically, circ_16601 functioned as a competitive sponge for miR-5580-5p, leading to the stabilization of *FGB* mRNA and subsequent upregulation of FGB expression. Ultimately, this process resulted in the activation of the Hippo/YAP1 pathway in vitro and in vivo.

## Methods

### Human samples

All the clinical specimen were approved by the Ethics Committee of the Second Hospital of Shandong University (approval number: KYLL-2020(KJ)P-0099). 7 pairs of tumor tissues and adjacent normal tissues were obtained from lung adenocarcinoma (LUAD) patients who had not received any chemotherapy or radiotherapy treatment before surgery at The Second Hospital of Shandong University between July 2019 and August 2020 (this study has been obtained written informed consent). A tissue microarray (TMA) containing 88 pairs LUAD samples and adjacent normal tissue samples were collected from 2016 to 2019 at The Pathology Department of Second Hospital of Shandong University. Two pathologists independently diagnosed and immediately stored all tissues in liquid nitrogen after surgical resection. The postoperative stage was determined based on the 8th edition of the International Union Against Cancer (UICC) tumor-node-metastasis (TNM) classification criteria.

### RNA-seq analysis

Total RNA extraction was used by TRIzol reagent (Invitrogen, USA), and RNA sequencing was performed by Novogene, China, on A549 vector cells (n = 3), A549 circ_16601_OE cells (n = 3), and A549 konckdown circ_16601#2/#3 cells (n = 3). Read counts were then subjected to RPM (Reads per million mapped reads) of each gene by TopHat-Cufflinks software. The DEG was performed with the R packages “DRseq2”. KEGG Function enrichment analysis of DEGs was performed with the R package “clusterProfiler”.

### Cell culture and transfection

Human Normal Bronchial Epithelial Cells 16-HBE and Beas-2B as well as human lung cancer cells A549, NCI-H1650, NCI-H520, NCI-H1299, and HEK-293T Cells were obtained from FUHENG Bio. Company (Shanghai, China). The 16-HBE, A549, NCI-H520, NCI-H1650, and NCI-H1299 cells were cultured in 1640 medium (Corning Cellgro, Manassas, VA, USA), while the HEK-293T cells were maintained in DMEM with high glucose (Corning, Manassas, VA, USA). All media were supplemented with fetal bovine serum (FBS, ExCellBio, Suzhou, China) at a final concentration of 10% and a final concentration of penicillin/streptomycin (Beyotime, Beijing, China) at 1%, All cells were maintained at 37 °C with 5% CO_2_. Stable transfection was performed using polybrene (10 mg/ml) (Solarbio, Beijing, China) based on the manufacturer's protocol. Stable cell lines were supplemented with a medium containing 1 μg/L puromycin (Beyotime, Shanghai, China) for 1–2 weeks.

### Plasmid and lentivirus construction

The miR-5580-5p mimics and inhibitor were synthesized from Tsingke Bio (Qingdao, China). The human FGB 3′-UTR WT/MUT luciferase reporter was obtained from Boshang Bio (Shanghai, China). Small interfering RNAs targeting FGB were also obtained from Tsingke Bio (Qingdao, China). The lentiviral plasmid for circ_16601 overexpression was constructed using the pLC5-ciR vector by Geneseed Bio. (Guangzhou, China). Meanwhile, the Crispr/Cas13 was used to knockdown circ_16601, sgRNA sequences targeting the back-splice site of circ_16601 were cloned in CRISPR-Cas13 vector. The shRNA targeting anti-YAP1 gene was ligated into the PLENT-U6-GFP-Puro vector, and the shRNA targeting FGB mRNA was ligated into the pLKO.1-Hygro lentivirus vector to construct the knockdown plasmid. To construct the stable miR-5580-5p overexpression plasmid, the sequence was cloned into the PCDH-copGFP-T2A vector (Tsingke Bio, Beijing, China). All plasmids were confirmed sequence by Sanger sequencing. The shRNA sequences are provided in Additional file 1: Table S2.

### Extraction of exosomes

As described in our previous report [31], the cells were spread into a 10 cm^2^ cell culture dish and changed to serum-free medium when the cell density reached 70–80%, subsequently the supernatant was collected after 48 h, the pellet was first discarded by centrifugation at 300*g* for 10 min, followed by centrifugation at 2000*g* for 10 min, followed by centrifugation at 1000*g* for 30 min, and finally exosomes were collected by centrifugation at 10,000*g* for 70 min with 1 × PBS.

### RT‒qPCR

Total RNA extraction was according to the standard protocol isolated using TRIzol reagent (Invitrogen, USA). RNA sequencing of A549 vector cells (n = 3), A549 circ_16601-overexpression cells (n = 3), and A549 circ_16601-knockdown cells (n = 3) was performed by Novogene (China). To test circRNA, mRNA, and miRNA expression, RT-qPCR were presented using the lnRcute lncRNA qPCR Kit and miRcute Plus miRNA qPCR Kit (SYBR Green, TIANGEN, Beijing, China) on the QuantStudio™ 5 RT-PCR System (Thermo Scientific, Carlsbad, CA, USA). ACTB or U6 was used as an internal control, and the relative expression of the target genes was calculated using the 2^−ΔΔCt^ method. The PCR primers of qPCR analyses are provided in Additional file 1: Table S3.

### Actinomycin D assays

A total of 2 × 10^5^ A549 and NCI-H1299 cells were sowed in 6-well plates/well for 24 h, and the cells were treated with 20 µM actinomycin D (Sigma) the next day. The cells were cracked at different time as shown in results, and RNA expression was evaluated via RT-qPCR. The values were normalized to the vehicle treatment group.

### Fluorescence in situ hybridization (FISH)

FISH assays were showed using RNA FISH kits (paraffin sections) (Gene Pharma, Shanghai, China) and Ribo™ FISH kits to observe the localization of circ_16601 in lung tissues and cells. Paraffin sections and cell slides were hybridized with a Cy3-specific labeled circ_16601 probe (Cy3-5′-CAA AAC UAU AAA GGA ACU GGC UUU AGA AAA GUA CUU UUC A-3′) (Qingke, Qingdao, China) according to the instructions. The cell slides were observed using an Olympus BX43 fluorescence microscope, and FISH images of paraffin sections were captured using a Nanozoomer digital pathological scanner.

### Immunohistochemistry (IHC) staining

Four μm paraffin-embedded tumor tissue sections were dewaxed and dehydrated after incubation at 65 °C for 1 h. Citric acid buffer (pH = 6) was used to boil the slides for 20 min at least. Endogenous peroxidase activity was blocked with 3% hydrogen peroxide at room temperature for 10 min. Normal goat serum was incubated in PBST at room temperature for 10 min, and the slides were incubated with primary antibodies against α-SMA (1:500, Proteintech, China), FGB (1:200, proteintech, China) and YAP1 (1:200, Proteintech, China) at 4 °C for 12–16 h. Rinse 3 times with PBS for 5 min each time, each section was incubated with the peroxidase labeled secondary antibody, developed with DAB IHC staining solution, and then counterstained with hematoxylin for 30 s to 2 min. The specimens were observed under a microscope, and the IHC pictures were gathered by Nanozoomer Digital Pathology scanner (NanoZoomer S60, Japan) and Inverted fluorescence microscope (Olympus IX73, Japan).

### Western blotting analysis

The experimental details were as previously reported [32]. Briefly, the cells lysis was pull out by RIPA lysis buffer, and 20 µg cell lysis was separated by 10% SDS-PAGE gel, and then the SDS gel was subsequently transferred to PVDF membranes (Millipore, Billerica, MA, USA). Primary antibodies against FGB (1:1000, Proteintech, China), YAP1 (1:1000, Proteintech, China), and ACTB (1:1000, Proteintech, China) were employed in the study.

### Soft agar assay

A bottom layer of 3 ml of 1% agar, prepared with full 2 × 1640 medium, was added, and the top layer was formed by pouring cell suspensions containing 5000 cells in 0.6% agar in complete medium. After 2–3 weeks, colonies were stained with crystal violet, and images were taken using a DMI8 inverted microscope (Leica Microsystems, Wetzlar, Germany). All data are presented as the mean ± SD, and each experiment was repeated at least three times.

### Luciferase reporter assay

The experimental details were as previously reported [32]. HEK-293T cells were seeded at a density of 5 × 10^4^ cells in 12-well plates and cotransfected with a mixture of 1 µg of pRL-TK, FGB-WT/MUT plasmids, and miRNA mimics. After 24 h of incubation, the luciferase enzyme activities were performed using a dual-luciferase reporter assay kit (Promega, Madison, WI, USA) and the data were measured via Cytation^5^ Cell Imaging Multi-Mode Reader (BioTek Instruments, Winooski, VT, USA).

### RNA pull-down assay

The RNA pull-down kit (Gene Seed Bio, Guangzhou, China) was performed to test the interaction between circ_16601 and miR-5580-5p. Biotinylated circ_16601 probes (CAA AAC UAU AAA GGA ACU GGC UUU AGA AAA GUA CUU UUC A), synthesized by Tsingke (Qingdao, China). 1 × 10^7^ cells were washed in pre-cold PBS, lysed in 1 ml buffer supplemented with 1% cocktail, 1 M DTT, and 1U/ul RNase inhibitor. The biotin-circ_16601/NC probes mixed the streptavidin magnetic beads pre-incubated at room temperature for 2 h and then incubated with cell lysate overnight at 4 ℃. The magnetic beads complex was washed with lysis buffers for 10 times. Finally, the purificated RNAs by RNAclean Kit (TIANGEN BIOTECH (BEIJING) CO., LTD) were used for RT-qPCR. The expression level of miRNA was confirmed using the miRcute Plus miRNA qPCR Kit (TIANGEN, Beijing, China) through RT‒qPCR.

### Xenograft model in nude mice in vivo

Fifty-four male BALB/c nude mice, 4 weeks old, were obtained from Vital River Research Animal Services in Beijing, China and were housed under specific-pathogen-free (SPF) conditions. Metastatic cancer models were established following a previously described method [33]. For the xenograft model, mice were allowed to acclimate for 1 week before being randomly assigned to one of three groups. A subcutaneous injection of 4 × 10^6^ cells was administered into the left flanks of the mice. Tumor volume was measured every 3 days after it reached 50 mm^3^. After 5 weeks, the mice were sacrificed, and one part of the tumors were fixed into 10% paraformaldehyde buffer for further analysis, while the other part was stored at − 80 ℃ for future use [32]. All animal experimental protocols were approved by the medical ethics committee of the Second Hospital of Shandong University (approval number: KYLL-2020(KJ)A-0138).

### Statistical analysis

The statistical analysis of the data was conducted using GraphPad 8.0 software, with Student's *t*-test used to determine significant differences between groups. A *p* value less than 0.05 was considered statistically significant.

## Results

### Upregulation of circ_16601 was widely observed in LUAD tissues and cell lines

**Fig. 1:**
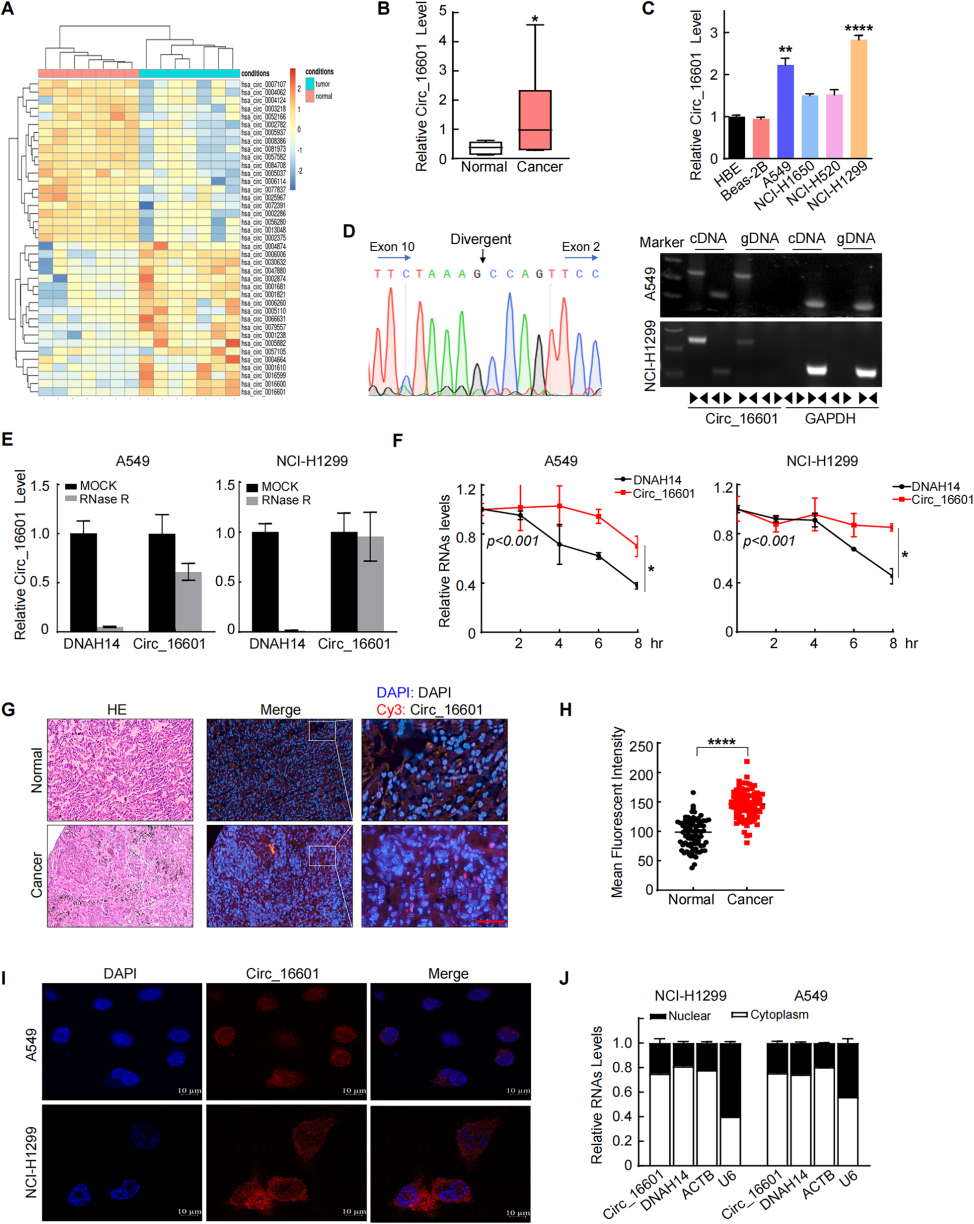
CircRNA expression profiles in LUAD and characterization of circ_16601. **A** A heatmap shows 39 differentially expressed circRNAs in seven tissue pairs from patients with LUAD; **B**, **C** Circ_16601 is upregulated in LUAD tissues and cell lines compared with normal LUAD tissues and cell lines (*P* < 0.05); **D** The back-splice junction site of circ_16601 was confirmed by RT–PCR followed by Sanger sequencing; agarose gel electrophoresis showed that circ_16601 was a circRNA, and it was amplified by diverging primers in cDNA but not in gDNA. GAPDH was used as a negative control; **E** The expression levels of circ_16601 and linear DNAH14 mRNA were determined by RT‒qPCR after RNase R treatment; **F** The expression levels of circ_16601 and linear DNAH14 mRNA were determined by RT‒qPCR after ACTD (20 µM) treatment; **G**, **H** Fluorescence in situ hybridization (FISH) confirmed circ_16601 expression in 7 paired LUAD samples by tissue microarrays; **I**, **J** Circ_16601 was mainly located in the cytoplasm, as confirmed by fluorescence in situ hybridization (FISH). Scale bars: 10 μm. *P < 0.05; **P < 0.01; ***P < 0.001; ****P < 0.0001

The present study focused on investigating the association between noncoding RNAs (ncRNAs) and cancer, with a focus on improving our understanding of this intricate relationship. Specifically, to elucidate the differential expression of circular RNA (circRNA) genes in LUAD tumors, RNA-seq (rRNA depletion) was performed on a total of 7 LUAD tissues and 7 adjacent normal tissues. Employing threshold criteria of a fold change > 3.0 and an adjusted* p* value < 0.05, we identified 21 upregulated circRNAs and 20 downregulated circRNAs (Additional file 1: Table S1). Notably, circ_16601 (circBase ID: hsa_circ_001661) exhibited the most significant difference in expression, displaying the highest fold change and lowest p-value (Fig. 1A). To validate these findings, we used Sanger sequencing and DNA fragment gel electrophoresis, which confirmed the presence of circ_16601 and its covalently closed continuous loop structure in LUAD tissues (Fig. 1D and Additional file 2: Fig. S1A). Furthermore, we observed overexpression of circ_16601 in LUAD tissues compared with normal tissues, and consistent results were observed in cells (Fig. 1B, C). It is worth noting that circ_16601 was resistant to RNase R enzyme digestion (Fig. 1E), and its expression was not affected by ACTD treatment (Fig. 1F) in the A549 and NCI-H1299 LUAD cell lines, which strongly suggests that circ_16601 possesses unique characteristics that distinguish it from other RNA species. Additionally, circ_16601 was found to be upregulated in LUAD tissue microarrays (TMAs) (Fig. 1G, H), and circ_16601 upregulation was more profound in patients with lymph node metastases or high T grades (Additional file 2: Fig. S1C). The association between the clinicopathological characteristics of 88 LUAD patients and circ_16601 expression is presented in Additional file 1: Table S1, indicating a correlation between circ_16601 expression and the T grades of the patients. Further investigations employing RNA fluorescence in situ hybridization (RNA-FISH) and nuclear and cytoplasmic reverse transcription quantitative PCR (RT‒qPCR) revealed that circ_16601 was primarily expressed in the cytoplasm of LUAD cells (Fig. 1I, J). Collectively, these results support the notion that circ_16601 is up-regulated in the cytoplasm of both LUAD tissues and cells, suggesting a potential role for circ_16601 in LUAD tumorigenesis.

### Circ_16601 promoted LUAD proliferation and invasion in vitro and in vivo

**Fig. 2:**
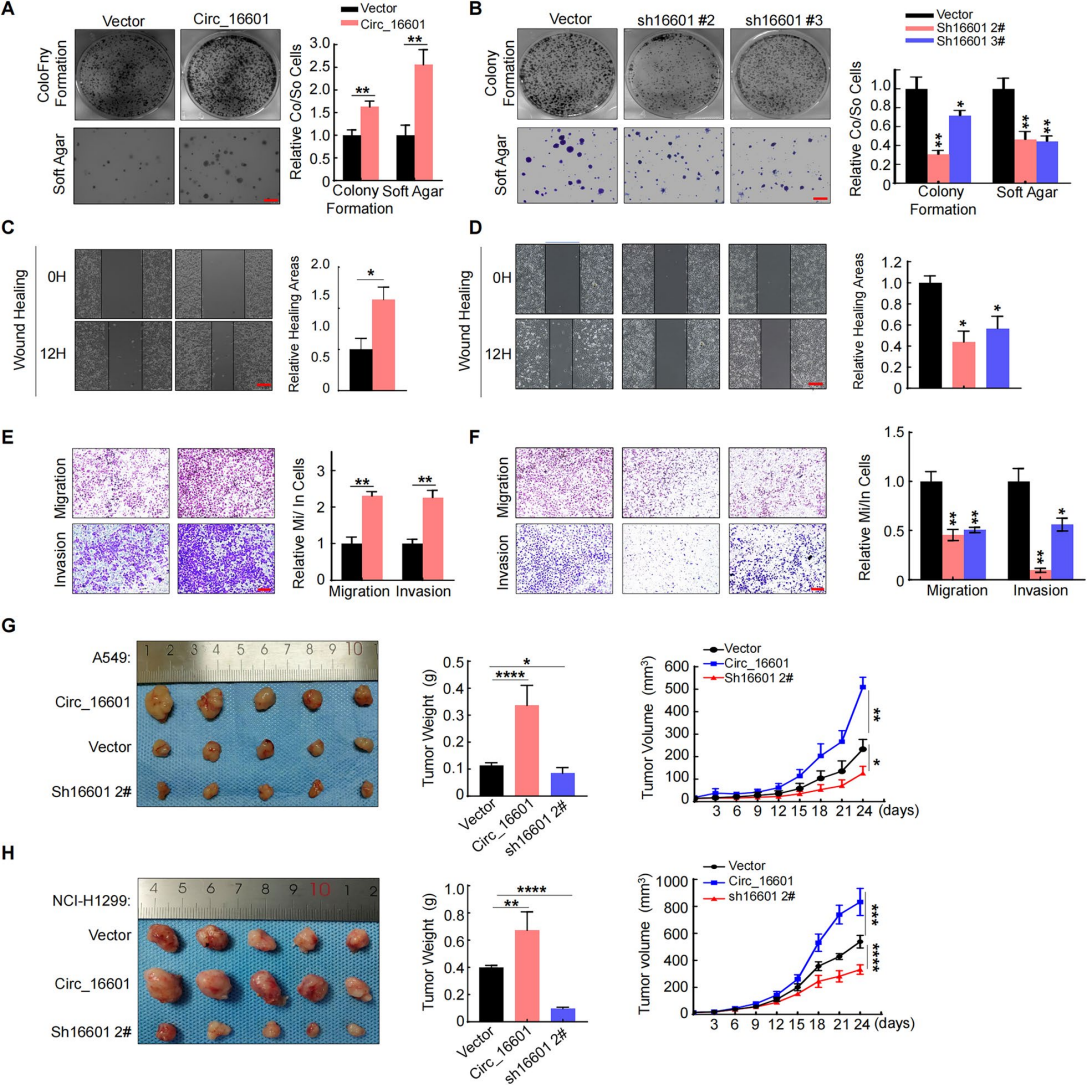
Circ_16601 promotes LUAD cell progression in vitro and in vivo. **A**, **C** Colony formation, and wound healing assays revealed that circ_16601 significantly accelerated the proliferation of A549 cells; **B**, **D** Colony formation, and wound healing assays revealed that circ_16601 knockdown significantly reduced proliferation in A549 cells; scale bars: 250 μm; **E**, **F** The migratory and invasive capacities of A549 cells transfected with the indicated virus were determined by Transwell assays. Scale bars: 250 μm; **G**, **H** The volume and weight of subcutaneous xenograft tumors (n = 5 mice per group). *P < 0.05; **P < 0.01; ***P < 0.001

To further investigate the functional and mechanistic role of circ_16601 in LUAD, an ectopic circ_16601 overexpression construct was prepared with the pLC5-ciR vector, as shown in Additional file 2: Fig. S2A, C, and shRNAs targeting circ_16601 were constructed with the pLenti-U6 vector, as shown in Additional file 2: Fig. S2B, D. In the soft agar assay, which confirmed the role of circ_16601 in facilitating anchorage-independent growth ability, and the colony formation experiment obtained the same conclusion for circ_16601 in LUAD cells (Fig. 2A, B and Additional file 2: Fig. S2E–H). Wound healing assays also confirmed the function of circ_16601 in cell migration (Fig. 2C, D and Additional file 2: Fig. S2I, J). Similarly, Transwell assays demonstrated that circ_16601 promoted LUAD cell migration and invasion ability (Fig. 2E, F and Additional file 2: Fig. S2K, L). Moreover, in BALB/c nude mice, circ_16601 overexpression significantly enhanced tumor growth in terms of both tumor weight and volume, while shRNA-mediated knockdown of circ_16601 abolished tumor growth (Fig. 2G, H). Collectively, these findings provide compelling evidence that circ_16601 plays an oncogenic role by promoting LUAD proliferation, migration, and invasion in both in vitro and in vivo settings.

### Circ_16601 accelerated my-CAF formation in the LUAD tumor microenvironment

**Fig. 3:**
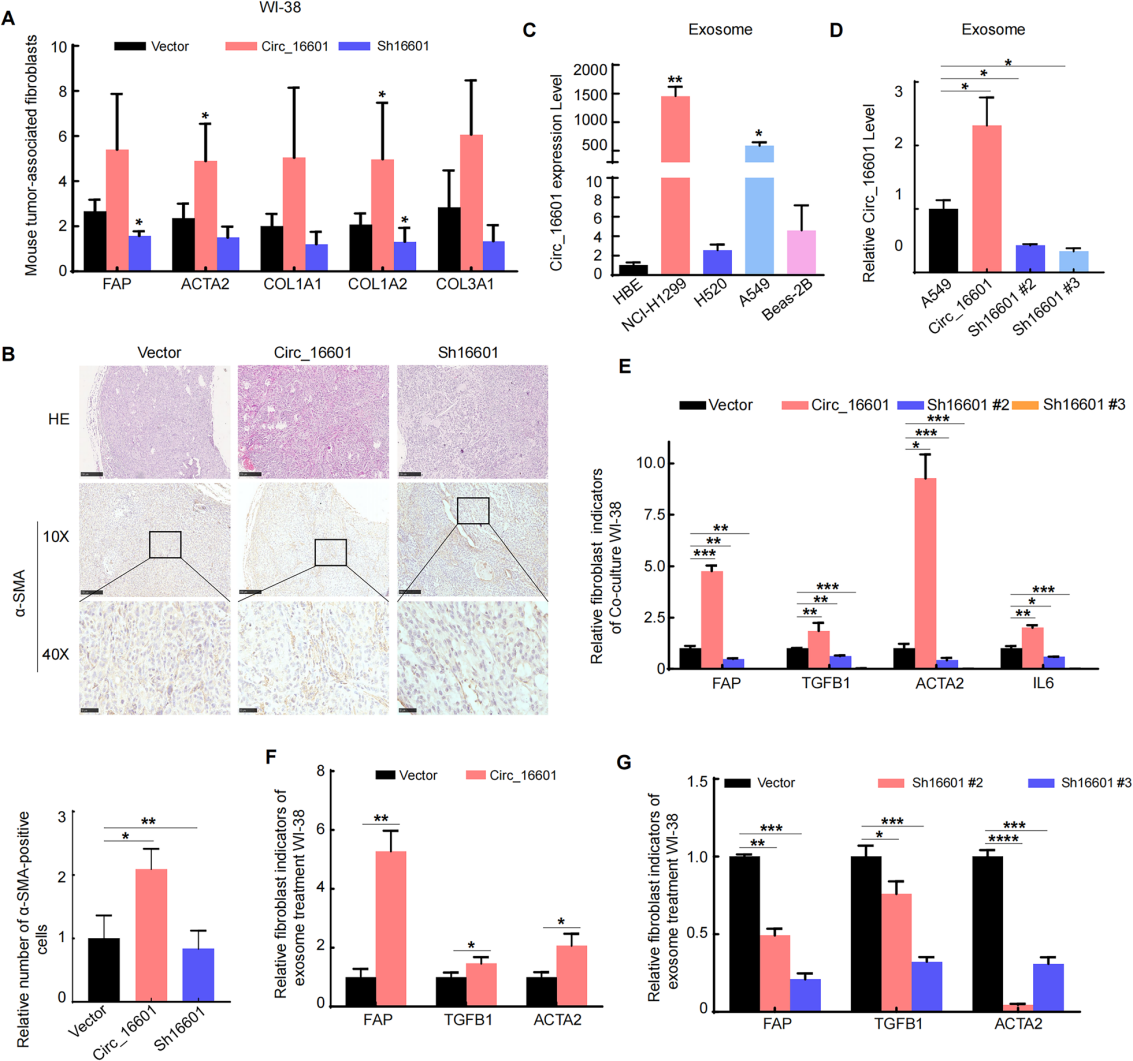
Exosome circ_16601 promotes my-CAF formation in the LUAD TME. **A** The effect of circ_16601 on the expression of tumor fibroblast markers was determined by analyzing mouse RNA with RT‐qPCR (n = 5 mice per group); **B** Representative IHC staining for α-SMA in subcutaneous tumors from nude mice; **C** RT‒qPCR assay was used to measure the expression level of exosomal circ_16601 from LUAD cell lines; **D** Expression of circ_16601 in secreted exosomes from A549 cells after circ_16601 knockdown or overexpression; **E** My-CAF protein marker expression was measured by RT‐qPCR after the co-culture with WI-38 cells; **F**, **G** My-CAF protein marker expression was measured by RT‐qPCR after WI-38 cells were treated with exosomes from circ_16601-knockdown or circ_16601-overexpressing A549 cells. *P < 0.05; **P < 0.01; ***P < 0.001; ****P < 0.001

It is widely known that CAFs are a vital component of the TME. The data described above demonstrated that circ_16601 overexpression increased LUAD progression; nevertheless, the mechanism and specific characteristics of circ_16601 that affect TME alteration should be further explored. Thus, we further investigated the expression of key CAF genes in mouse xenograft tumors using reverse transcription quantitative PCR (RT-qPCR) (Fig. 2G). As shown in Fig. 3A, the my-CAF marker genes *ACTA2*, *FAP*, *COL1A1*, *COL1A2*, and *COL3A1* were slightly upregulated after circ_16601 overexpression and downregulated after circ_16601 knockdown in xenograft tumors. The IHC results in Fig. 3B were consistent with the PCR results, demonstrating the role of circ_16601 in promoting my-CAF recruitment in LUAD. Numerous studies have reported that circRNAs can be packaged to regulate adjacent cells in the TME [34, 35]. To determine whether circ_16601 could be released outside of cells, we first measured the level of circ_16601 in LUAD cell lines. The results showed that circ_16601 was not only secreted by exosomes but also expressed at higher levels in tumor cell-derived exosomes compared to normal lung epithelial cell-derived exosomes (Fig. 3C). Furthermore, we also found that circ_16601 was upregulated in the exosomes from circ_16601-overexpressing A549 cells, while it was downregulated in the exosomes from circ_16601-knockdown A549 cells (Fig. 3D). To verify the hypothesis that circ_16601 promotes my-CAF formation in LUAD via exosome secretion, a cell culture system was established in which WI-38 cells were co-cultured with circ_16601-overexpressing and circ_16601-knockdown cells. As shown in Fig. 3E, my-CAF marker genes (*ACTA2, FAP*, *TGFB1*, and *IL6*) were markedly increased in WI-38 fibroblast cells, but after circ_16601 knockdown in A549 cells, completely opposite results were observed. Moreover, exosomes were obtained from A549 cells (vector, circ_16601-overexpression, circ_16601-knockdown) by ultracentrifugation, as shown in Fig. 3F and G, and exosomes derived from circ_16601-overexpressing cells firmly increased the *ACTA2*, *FAP*, and *TGFB1* mRNA levels, while circ_16601-konckdown markedly decreased the *ACTA2*, *FAP*, and *TGFB1* mRNA levels. Taken together, these data strongly suggest that circ_16601 effectively influences elements of the TME by promoting my-CAF formation.

### Circ_16601 functions as a sponge for miR-5580-5p to promote progression in LUAD

**Fig. 4:**
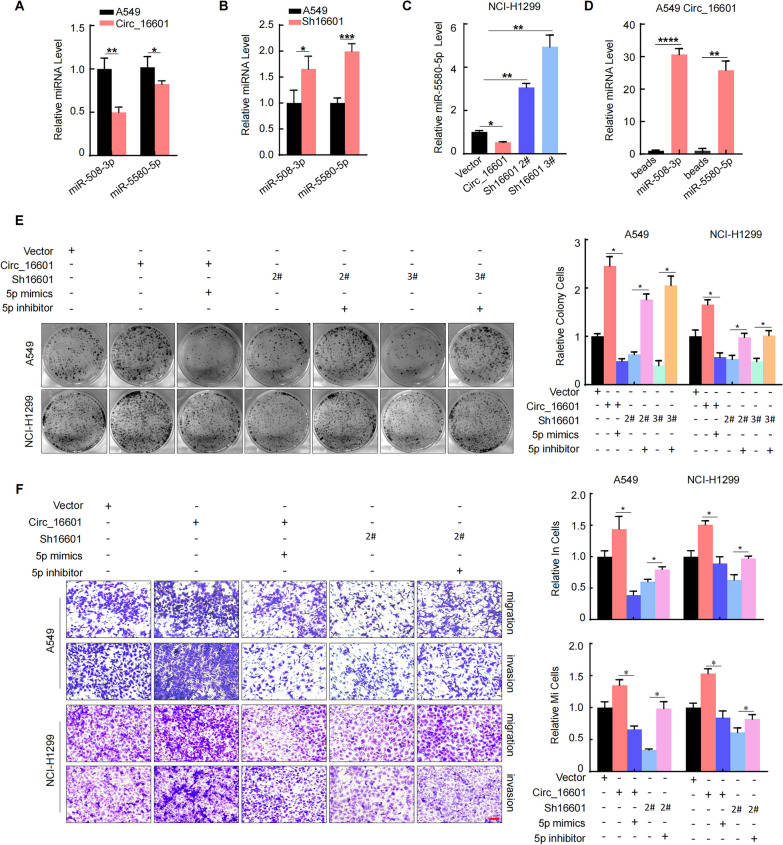
Circ_16601 acts as a miR-5580-5p sponge to promote LUAD progression. **A**, **B** Relative expression of miR-5580-5p and miR-508-3p in A549 cells after circ_16601 overexpression or knockdown; **C** Relative expression of miR-5580-5p in NCI-H1299 cells after circ_16601 overexpression or knockdown; **D** RIP assay for circ_16601 was performed, and the coimmunoprecipitated RNA was subjected to RT‒qPCR to measure miR-5580-5p and miR-508-3p expression; **E**, **F** Colony formation, Transwell assays were performed to determine the proliferation and invasion ability of A549 cells after treatment. Scale bars: 100 μm. *P < 0.05; **P < 0.01; ***P < 0.001; ****P < 0.001

It is well known that circRNA can play a biological role by adsorbing miRNA. To further explore the miRNAs that directly sponged by circ_16601, we screened 29 common miRNAs by screening the circBank and miRWalk databases (Additional file 2: Fig. S3A and Additional file 1: Table S4). RT‒qPCR results further showed that circ_16601 inhibited only two miRNAs, namely, miR-508-3p and miR-5580-5p, in A549 cells (Fig. 4A and Additional file 2: Fig. S3B, C), and circ_16601-konckdown upregulated miR-508-3p and miR-5580-5p expression simultaneously (Fig. 4B). RNA pull-down assays confirmed the direct binding of circ_16601 to miR-5580-5p in LUAD cells (Fig. 4D). However, we found it intriguing that only miR-5580-5p regulation was observed in NCI-H1299 cells (Fig. 4C and Additional file 2: Fig. S3D). To determine whether miR-5580-5p functions downstream of circ_16601 in LUAD cells, miR-5580-5p mimics were transfected into circ_16601-overexpressing cells, and a miR-5580-5p inhibitor was transfected into circ_16601-knockdown cells. Colony formation and Transwell assays consistently demonstrated that miR-5580-5p mimics reversed the functional effects of circ_16601 in promoting LUAD progression (Fig. 4E, F). Collectively, these results indicate that circ_16601 binds to miR-5580-5p and subsequently enhances progression in LUAD cells.

### Circ_16601 specifically stabilized FGB mRNA by binding to miR-5580-5p in LUAD cells

**Fig. 5:**
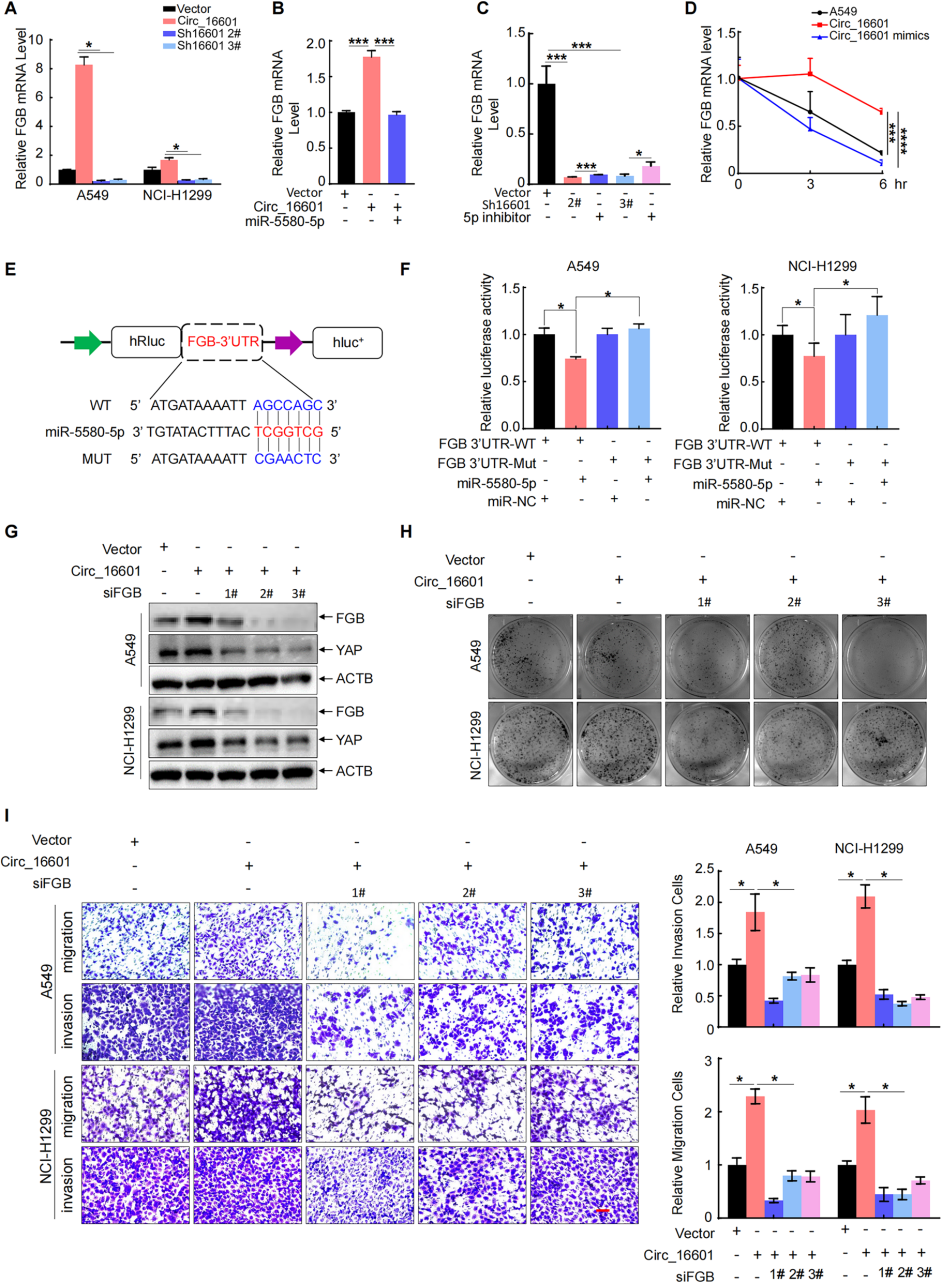
FGB is downstream of the circ_16601/miR-5580-5p complex in LUAD. **A** The mRNA expression of *FGB* in A549 and NCI-H1299 cells was measured by RT‐qPCR; **B** Relative mRNA expression of *FGB* in A549 cells overexpressing circ_16601 after treatment with miR-5580-5p mimics; **C** Relative mRNA expression of *FGB* in circ_16601-knockdown A549 cells after treatment with the miR-5580-5p inhibitor; **D** The stability of *FGB* mRNA was verified by ACTD (20 µM) assay; **E**, **F** An illustration of the wild-type sequence and mutant sequence of the binding site of miR-5580-5p in the *FGB* promoter region is shown. Relative luciferase activities were measured in A549 cells after transfection with luciferase reporter plasmids carrying the wild-type *FGB* promoter domain, mutant *FGB* promoter domain, or negative control. **G** The protein expression of FGB and YAP1 was measured by Western blot. **H** Colony formation assays revealed that FGB significantly accelerated the proliferation of A549 and NCI-H1299 cells. **I** The migratory and invasive capacities of A549 and NCI-H1299 cells transfected with siFGBs were determined by Transwell assays. Scale bars: 100 μm. *P < 0.05; **P < 0.01; ***P < 0.001; ****P < 0.001

It is well established that miRNAs are capable of negatively regulating the mRNA stability of their target genes [36]. In this study, we investigated the differentially expressed genes (DEGs) identified by RNA-seq upon circ_16601 overexpression and knockdown in LUAD cells and compared them with the target genes of miR-5580-5p from the miRWalk database. Among the overlapping genes, FGB was identified as a potential target of circ_16601 and miR-5580-5p (Additional file 2: Fig. S3F). To confirm the role of circ_16601 in regulating *FGB* mRNA expression, we examined the *FGB* mRNA levels in circ_16601-overexpressing or circ_16601-knockdown A549 and NCI-H1299 cells. As demonstrated in Fig. 5A, the *FGB* mRNA levels were found to be elevated after circ_16601 overexpression and inhibited after circ_16601 knockdown. This result confirmed that circ_16601 positively regulates *FGB* mRNA expression. In addition, we found that miR-5580-5p mimics significantly inhibited the *FGB* mRNA levels, while a miR-5580-5p inhibitor upregulated the *FGB* mRNA levels in circ_16601-knockdown cells (Fig. 5B, C and Additional file 2: Fig. S3E). Moreover, circ_16601 overexpression stabilized *FGB* mRNA, and this stability was significantly abolished by miR-5580-5p mimics (Fig. 5D). To further confirm the role of miR-5580-5p in regulating *FGB* 3′-UTR activity, we generated a mutation in the binding site between *FGB* and the miR-5580-5p luciferase reporter in the pmiR-reporter backbone plasmid (Fig. 5E). The luciferase reporter assay in Fig. 5F confirmed that miR-5580-5p indeed disrupted *FGB* mRNA stability and luciferase reporter gene activity. These results provide strong evidence for the existence of a circ_16601/miR-5580-5p/FGB regulatory axis. Furthermore, to better understand the functional role of *FGB* downstream of circ_16601, we used siRNA to knock down *FGB* in cells overexpressing circ_16601 (Fig. 5G). Notably, the knockdown of FGB significantly abrogated the oncogenic effects induced by circ_16601 overexpression, as evidenced by the reduction in cellular proliferation, migration, and invasion (Fig. 5H,I). These findings highlight the pivotal contribution of FGB to the regulatory axis and extend our understanding of the complex interplay among circ_16601, miR-5580-5p, and FGB in cancer development.

### Circ_16601 activated the Hippo pathway via elevated YAP1 expression in LUAD cells

**Fig. 6:**
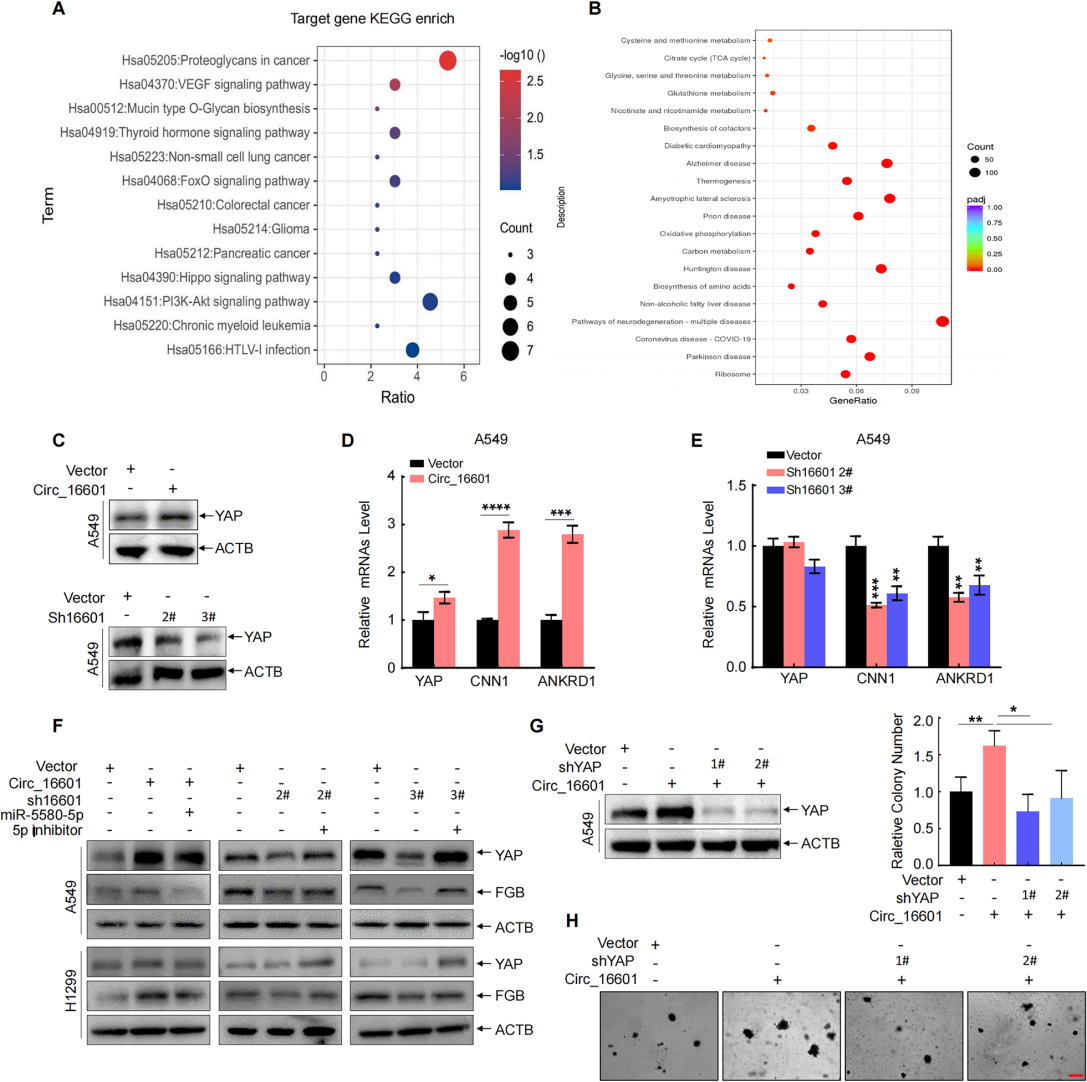
Circ_16601 upregulates YAP1 expression in LUAD. **A**, **B** KEGG pathway analysis of differentially expressed genes. The bubble chart shows the enrichment of differentially expressed genes in signaling pathways. The size and color of the bubble represent the number of differentially expressed genes enriched in the pathway and enrichment significance, respectively. **C** Western blotting was performed to measure the expression of the YAP1 protein. **D**, **E** The expression of genes downstream of YAP1 in A549 cells was measured by RT‐qPCR after the overexpression or knockdown of circ_16601. **F** Western blotting was performed to measure the expression of the YAP1 and FGB proteins in A549 and NCI-H1299 cells. **G** Western blotting was performed to measure the expression of the YAP1 protein in A549 cells after overexpression of circ_16601 and transfection with shYAP 1#/2#. **H** Soft agar assays were performed to verify the proliferation and malignancy of A549 cells after overexpression of circ_16601 and transfection with shYAP 1#/2#. *P < 0.05; **P < 0.01; ***P < 0.001; ****P < 0.001

The previous section of this study established that circ_16601 acts as a sponge for miR-5580-5p, leading to the downregulation of miR-5580-5p expression and negatively regulating the mRNA stability of its target gene *FGB*, ultimately promoting LUAD cell progression. To elucidate the underlying mechanisms by which circ_16601 regulates signaling pathways, we utilized RNA-seq and performed KEGG and GSEA analyses. These analyses revealed that circ_16601 activates the Hippo pathway and that the inhibition of circ_16601 leads to a decrease in Hippo pathway activation (Fig. 6A and B). Of note, *YAP1*, which is a key gene of the Hippo pathway, exhibited changes in expression that were consistent with the changes in circ_16601 expression (Fig. 6C). Furthermore, downstream target genes of *YAP1*, such as *CNN1*, and *ANKRD1*, were upregulated in circ_16601-overexpressing cells and downregulated in circ_16601-knockdown cells (Fig. 6D and 6E). To investigate whether the activation of the Hippo pathway is regulated by the previously demonstrated circ_16601/miR-5580-5p/FGB regulatory axis, we transfected A549 cells with miR-5580-5p mimics and inhibitors after the overexpression or knockdown of circ_16601, as shown in Fig. 6F. We also inhibited the Hippo pathway by knocking down YAP1 in circ_16601-overexpressed A549 cells (Fig. 6G). Notably, the knockdown of YAP1 abrogated the oncogenic effects of circ_16601 overexpression, as evidenced by the reduction in cellular proliferation (Fig. 6H). Collectively, these findings strongly support the pivotal role of circ_16601 in modulating the Hippo signaling pathway, ultimately promoting malignant properties in LUAD.

### The circ_16601/miR-5580-5p/FGB regulatory axis was confirmed in vivo

**Fig. 7:**
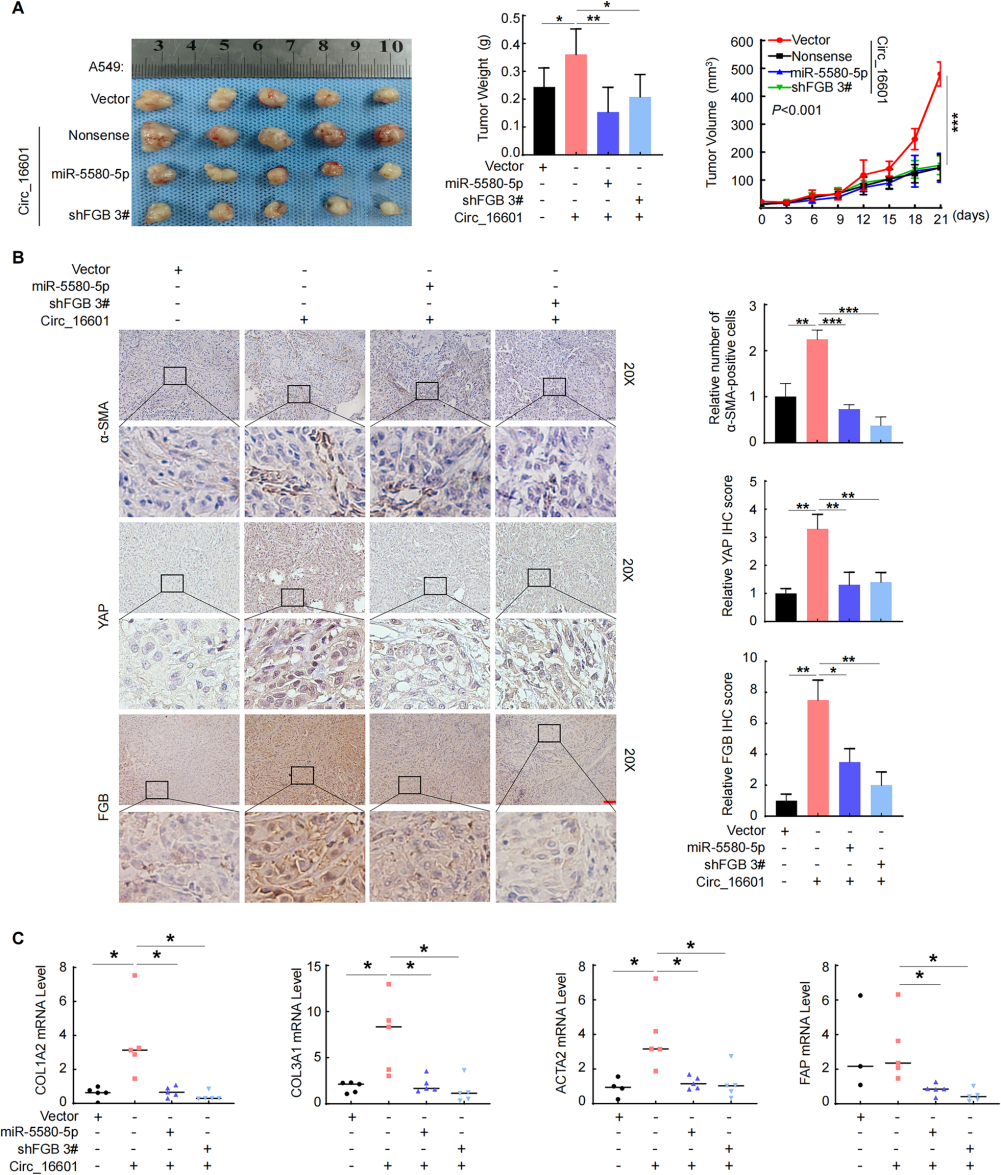
The circ_16601/miR-5580-5p/FGB regulatory axis was confirmed in vivo. **A** The volume and weight of subcutaneous xenograft tumors (n = 5 mice per group); **B** Immunohistochemistry (IHC) staining for α-sma, FAP, FGB, and YAP1 in tumors from mice. Scale bars: 100 μM. Three independent experiments were conducted; **C** RT‐qPCR was performed to measure the expression levels of *α-SMA*, *FAP*, *FGB*, and *YAP1* in tumors from mice. *P < 0.05; **P < 0.01; ***P < 0.001

After the aforementioned investigation, we reached the firm conclusion that circ_16601 functions by enhancing *FGB* mRNA stability through miR-5580-5p sequestration, which in turn activated the Hippo pathway. However, it was imperative to corroborate these findings in vivo. To this end, we established an A549 cell xenograft mouse model, as illustrated in Fig. 7A. As expected, overexpression of circ_16601 in A549 cells significantly accelerated tumor growth in the xenograft mouse model. However, overexpression of miR-5580-5p and knockdown of FGB effectively reversed the tumor-promoting effect of circ_16601 in LUAD, as evidenced by measuring tumor weight and volume. Moreover, the soft agar experiment also yielded consistent results (Additional file 2: Fig. S3G). Additionally, we confirmed the expression of genes that are downstream of circ_16601, namely, *FGB*, *YAP1*, and *ACTA2*, using an immunohistochemistry (IHC) assay (Fig. 7B). Consistent with the in vitro results, these findings further suggest a role for the circ_16601/miR-5580-5p/FGB regulatory axis in LUAD. Furthermore, we also evaluated the expression of myofibroblast marker genes, such as *COL1A2*, *COL3A1*, *ACTA2*, and *FAP*, in the tumor microenvironment using an IHC assay (Fig. 7C). Our results indicate that the circ_16601 regulatory axis not only activates the Hippo pathway but also regulates myofibroblast formation in the tumor environment. In summary, our study provides compelling evidence that circ_16601 plays a crucial role in modulating the tumor microenvironment, thus promoting the progression of LUAD.

## Discussion

**Fig. 8:**
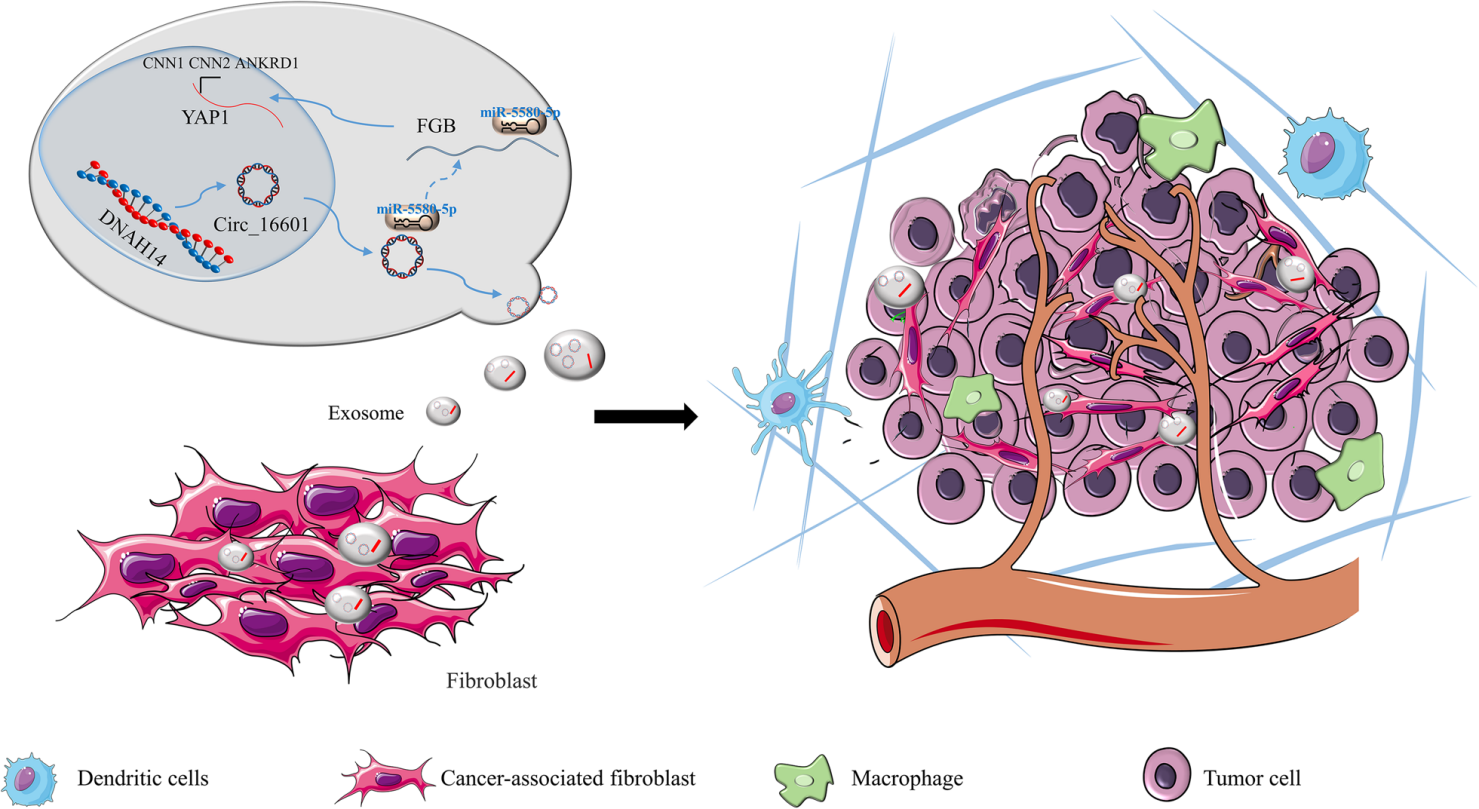
Model summarizing circ_16601 acted as a molecular sponge for miR-5580-5p to promote the FGB expression of the downstream target gene of miR-5580-5p, activated the Hippo pathway, and then affect fibroblasts in the tumor microenvironment

Recent research has shown a growing interest in investigating the potential roles of circRNAs in cancer, with a particular focus on their interaction within the tumor microenvironment (TME) [37–39]. The TME encompasses a complex network of diverse cell types, including immune cells, cancer cells, and stromal cells, along with extracellular matrix (ECM) components and various signaling molecules [40]. The TME plays a critical role in cancer progression and is a major determinant of therapeutic response [40]. Our study represents the first report that circ_16601 exerts an oncogenic effect on the TME via exosomes in LUAD (Fig. 8). Numerous studies have demonstrated the involvement of circRNAs in regulating the TME in various cancer types. For instance, circRNA CDR1-AS has been reported to be upregulated in colorectal cancer (CRC) [41]. CircRNA-MYLK was also shown to play a role in the regulation of the ECM in the TME [42]. In our study, circ_16601 was found to recruit my-CAFs, a key component of the TME, by upregulating the expression of α-SMA and collagen-related genes.

The distinctive circular conformation of circRNAs confers upon them a remarkable resilience against exonucleolytic degradation by RNase R, a phenomenon attributed to the circular formation of covalent bonds between their 3′ termini and 5′ terminal [43, 44]. Owing to this inherent stability, even a modest presence of circRNAs can wield substantial influence over key biological processes associated with the onset and advancement of tumorigenesis. In addition, miR-5580-5p has not been reported to interact with any circRNA, although various studies have found circRNA to act as adsorption sponges or chelates miRNAs to disrupt miRNA inhibition of target genes. Meanwhile, the role of miR-5580-5p in lung cancer is barely reported, and it was found only to work as a suppressor gene in oral cancer in publication [45]. Here, we provide the first evidence of the antitumor effect of miR-5580-5p and elucidate its detailed mechanism of action in LUAD. Overexpression of miR-5580-5p evidently abolished the growth and invasion promotion of circ_16601 in lung cancer cells. It was further found that miR-5580-5p targeted FGB and destroyed *FGB* mRNA stability, resulting in a decline in *FGB* mRNA. The findings in our study not only reveal the regulatory axis of the circ_16601/miR-5580-5p/FGB axis but also enrich the regulatory mechanism of circRNA. In light of the aforementioned insights, our results provide a novel perspective for a comprehensive understanding of the factors contributing to tumor heterogeneity. Beyond its role as a miRNA sponge, circRNA possesses the capacity to engage with RNA-binding proteins and, intriguingly, can serve as a template for peptide translation, thereby participating in multifarious biological functions. The putative involvement of circ_16601 in these mechanisms warrants further comprehensive investigation and elucidation.

FGB is a glycoprotein that is involved in blood clotting, and Hippo/YAP1, a regulator of cellular processes including proliferation, apoptosis, and differentiation [21, 46], have both been implicated in cancer development and progression. Increased expression of FGB has been associated with cancer metastasis and poor prognosis in various types of cancers, while YAP1 overexpression has been observed in multiple cancers, promoting tumor growth and metastasis. However, the regulatory connection between FGB and YAP1 has not been explored. In the present study, we uncovered that FGB could activate YAP1 expression and downstream genes of the Hippo pathway and suggest that circ_16601 activates the Hippo pathway via FGB upregulation. However, further research is needed to completely elucidate the mechanisms underlying the interaction among circ_16601, FGB, and the Hippo pathway and to explore the potential use of this interaction as a therapeutic target for various diseases.

## Conclusions

Our study provides compelling evidence for the oncogenic role of circ_16601 in promoting LUAD cell proliferation, migration, and invasion both in vitro and in vivo. Additionally, we demonstrate that exosomal circ_16601 promotes the recruitment of myoblast fibroblasts, fostering a cancer-promoting environment within the TME, thus contributing to the progression of LUAD. These findings underscore the significant involvement of circRNAs in cancer progression and suggest that targeting circRNAs may represent a promising therapeutic strategy for cancer treatment.

### Supplementary Information

**Additional file 1. **Supplementary Tables.

**Additional file 2.** Supplementary materials.

## Data Availability

The data supporting the findings of this study are available from the corresponding author upon reasonable request.

## Abbreviations

LUAD: Lung adenocarcinoma
CAF: Cancer associated fibroblasts
FGB: Fibrinogen beta chain
YAP1: Yes associated protein 1
TME: Tumor microenvironment
ACTA2/α-SMA: α-Smooth muscle actin
FAP: Fibroblast activation protein
TGFB1: TGF beta 1 protein
IL6: Interleukin 6
COL1A2: Collagen type I alpha 2 chain
COL3A1: Collagen type III alpha 1 chain
CNN1: Calponin 1
ANKRD1: Recombinant Ankyrin Repeat Domain Protein 1
