# Supplementary material for: Circ_16601 facilitates Hippo pathway signaling via the miR-5580-5p/FGB axis to promote my-CAF recruitment in the TME and LUAD progression
Source: Respir Res. 2023 Nov 12;24:276. doi: 10.1186/s12931-023-02566-4 (PMC10642073; doi:10.1186/s12931-023-02566-4)
Supplement: Supplementary file 2 — Additional file 2. Supplementary materials. [file 12931_2023_2566_MOESM2_ESM.pdf]

Figure S1

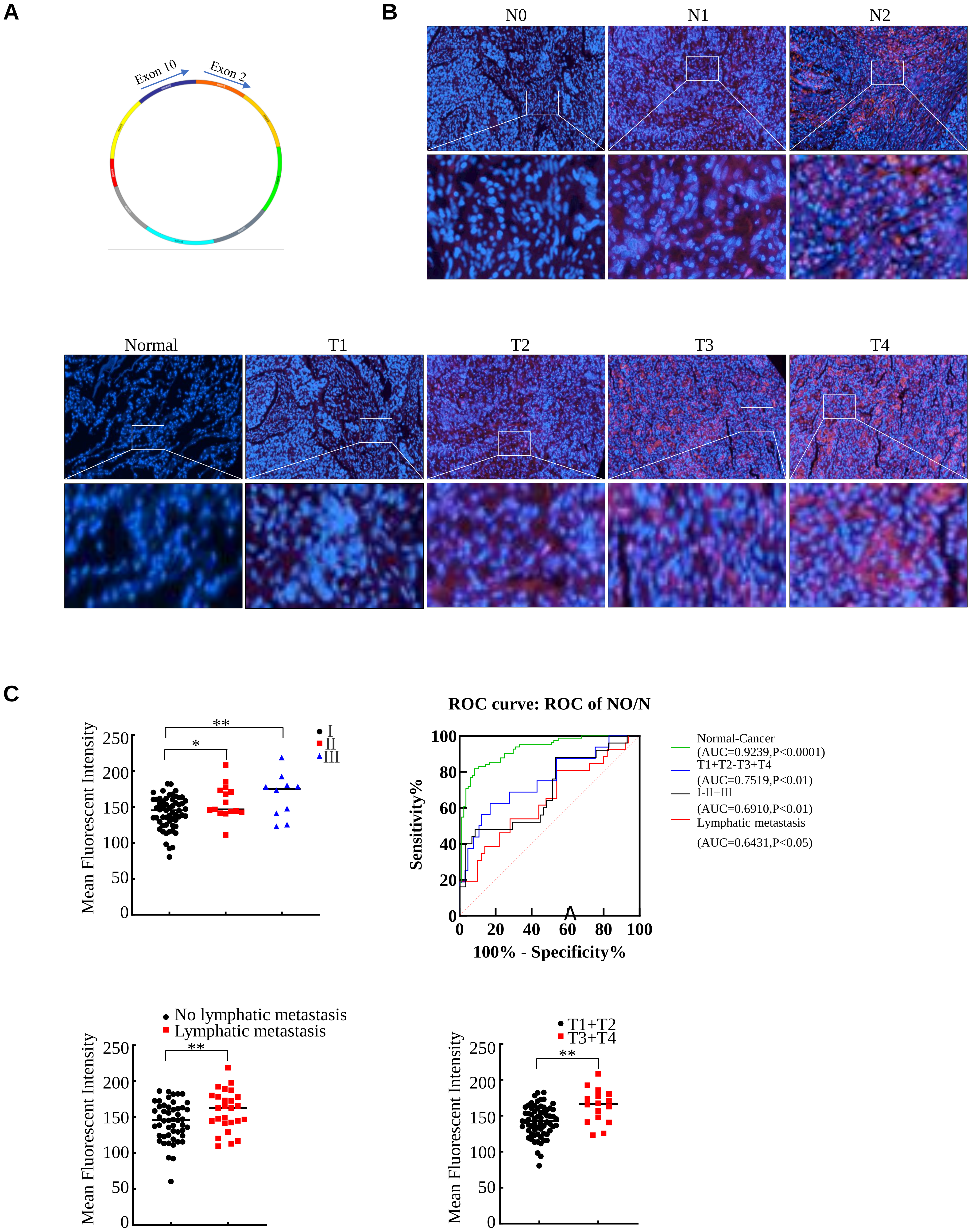

Supplemental Figure 1: (A) Schematic illustration of circ_16601. The genome structure shows that circ_16601 is cycled from the 2nd to 10th exons of the parent molecule DNAH14. (B, C) The relationship between circ_16601 and the pathological stage was determined by fluorescence in situ hybridization (FISH) of tissue chips. Scale bars: 100 µm. *$P < 0.05$; **$P < 0.01$

Figure S2

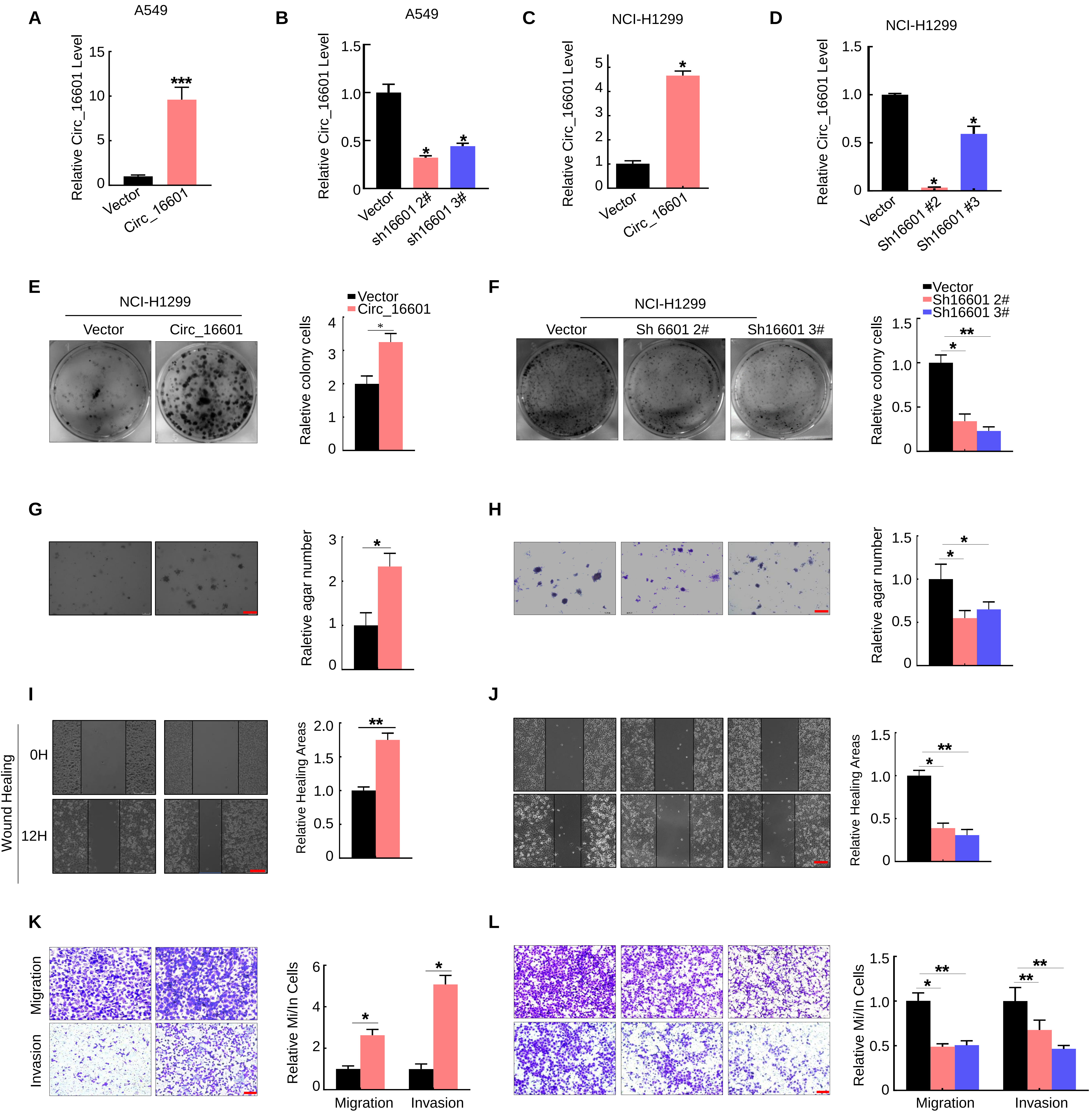

Supplemental Figure 2 Circ_16601 promotes LUAD cell progression. (A,C) RT-qPCR was used to determine the efficacy of circ_16601 overexpression in A549 cells and NCI-H1299 cells. (B, D) RT-qPCR was used to determine the efficacy of circ_16601 knockdown in A549 cells and NCI-H1299 cells. (E, G, I) Colony formation, soft agar and wound healing assays revealed that circ_16601 significantly accelerated the proliferation of NCI-H1299 cells. Scale bars: 250 μm. (F, H, J) Colony formation, soft agar and wound healing assays revealed that circ_16601 knockdown significantly reduced the proliferation of NCI-H1299 cells. (G, H) A soft agar assay was performed to verify the degree of malignancy in NCI-H1299 cells. (K, L) The migratory and invasive capacities of NCI-H1299 cells after transfection with the indicated virus were determined by Transwell assays. Scale bars: 250 μm. *P < 0.05; **P < 0.01; ***P < 0.01

Figure S3

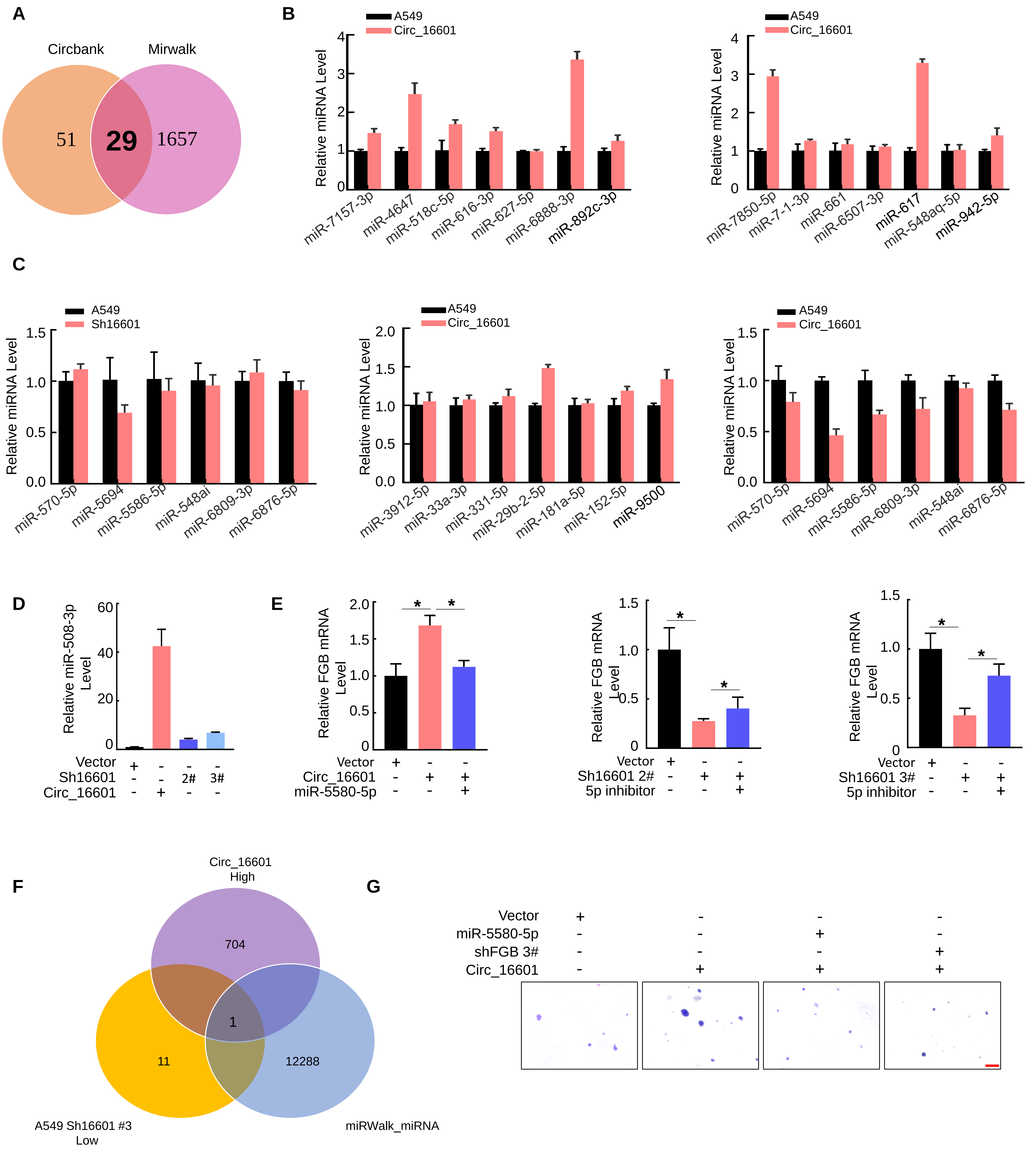

Supplemental Figure 3 (A) Schematic of the prediction that miRNAs can bind to circ_16601 according to the circBank and can bind to FGB mRNA according to miRWalk databases; (B, C) The expression of miRNAs in A549 cells was verified by RT-qPCR; (D) The expression of miR-508-3p in NCI-H1299 cells was verified by RT-qPCR; (E) Relative mRNA expression of *FGB* in NCI-H1299 cells after circ_16601 overexpression or knockdown and treatment with miR-5580-5p mimics or inhibitor; (F) The sequencing data of circ_16601-overexpressing and circ_16601-knockdown cells were intersected with the database miRWalk; (G) Soft agar assay was performed to verify the degree of malignancy in A549 cells. Scale bars: 250 μm.
